# Effect of MRE11 Loss on PARP-Inhibitor Sensitivity in Endometrial Cancer *In Vitro*

**DOI:** 10.1371/journal.pone.0100041

**Published:** 2014-06-13

**Authors:** Romana Koppensteiner, Eleftherios P. Samartzis, Aurelia Noske, Adriana von Teichman, Ioannis Dedes, Myriam Gwerder, Patrick Imesch, Kristian Ikenberg, Holger Moch, Daniel Fink, Manuel Stucki, Konstantin J. Dedes

**Affiliations:** 1 Division of Gynaecology, University Hospital of Zurich, Zurich, Switzerland; 2 Institute of Surgical Pathology, University Hospital of Zurich, Zurich, Switzerland; St. Georges University of London, United Kingdom

## Abstract

**Aim of the study:**

To evaluate the frequency of MRE11/RAD50/NBS1 (MRN)-complex loss of protein expression in endometrial cancers (EC) and to determine whether loss of MRE11 renders the cancer cells sensitive to Poly(ADP-ribose) polymerase (PARP)-inhibitory treatment.

**Methods:**

MRN expression was examined in 521 samples of endometrial carcinomas and in 10 cancer cell lines. A putative mutation hotspot in the form of an intronic poly(T) allele in MRE11 was sequenced in selected cases (n = 26). Sensitivity to the PARP-inhibitor, BMN673 was tested in colony formation assays before and after MRE11 silencing using siRNA. Homologous recombination (HR) DNA repair was evaluated by RAD51-foci formation assay upon irradiation and drug treatment.

**Results:**

Loss of MRE11 protein was found in 30.7% of EC tumours and significantly associated with loss of RAD50, NBS1 and mismatch repair protein expression. One endometrial cell line showed a markedly reduced MRE11 expression due to a homozygous poly(T) mutation of MRE11, thereby exhibiting an increased sensitivity to BMN673. MRE11 depletion sensitizes MRE11 expressing EC cell lines to the treatment with BMN673. The increased sensitivity to PARP-inhibition correlates with reduced RAD51 foci formation upon ionizing radiation in MRE11-depleted cells.

**Conclusion:**

Loss of the MRE11 protein predicts sensitivity to PARP-inhibitor sensitivity in vitro, defining it as an additional synthetic lethal gene with PARP. The high incidence of MRE11 loss in ECs can be potentially exploited for PARP-inhibitor therapy. Furthermore, MRE11 protein expression using immunohistochemistry could be investigated as a predictive biomarker for PARP-inhibitor treatment.

## Introduction

Endometrial cancer (EC) is the fourth most common malignancy among women. The majority of ECs are diagnosed in early stage and are associated with very favourable overall prognosis [1]. Treatment options, however, for advanced, recurrent or metastatic ECs, are limited and consist mainly of cytotoxic chemotherapy [1]. Potential targeted treatments are under clinical investigations but have not yet been incorporated in routine clinical use [2].

EC is a heterogeneous disease with distinct histological and molecular characteristics [2]. So far, EC have been classified into types I and II. This is based on the different histological properties (endometrioid vs. non-endometrioid) and on the clinical prognosis (favourable vs. poor) [2], [3]. In addition, distinct molecular alterations occur preferentially in either type I or type II EC (reviewed in [2]). Whereas type I tumours are characterized by microsatellite instability (MSI) and polymutations in different types of genes, almost all type II tumours harbour mutations of the tumour suppressor gene *TP53* [4]. Recently, novel molecular subgroups have been described in a way akin to breast cancer [5]. Based on their mutation profile and copy-number changes ECs are categorized into: the ultramutated, the hypermuted, the copy number low and the copy number high subgroup [5].

The hypermutated subgroup includes mostly endometrioid EC, all harbouring microsatellite instability (MSI). These tumours are known to develop mutations in various other genes (hypermutated genome) but also those involved in the DNA double strand break (DSB) repair machinery [6]. One of the most common recurrent mutation is found in the *MRE11 gene*, whose product is a part of the MRE11-RAD50-NBS1 (MRN)- complex that is involved in the detection and repair of DNA double-strand breaks (DSBs) [7], [8]. *MRE11* germline mutations that cause a lethal phenotype in mice [9] are rarely encountered in humans and lead to an Ataxia telangiectasia-like disorder (ATLD) [10]. Somatic mutations in *MRE11*, however, are frequently detected in colorectal cancers with MSI and have also been suggested for MSI-positive ECs [11]. Mutations of the intronic poly(T) sequence of *MRE11* between exons 4 and 5 are frequent events in MSI positive colorectal und ECs [11], [12]. In EC, MSI is present in more than 20% of tumours and is mainly caused by epigenetic silencing of the MMR gene *MLH1* [13]. This leads to changes in the number of nucleotide repeats found in coding and non-coding elements of many genes such as *MRE11* [14].

Synthetic lethality occurs when two individually occurring mutations have no effect on cell viability, but cause cell death in combination [15], [16]. Inhibition of a synthetic lethal partner gene in cancer cells presenting a synthetic lethal mutation may prove an attractive strategy to develop specific anti-cancer drugs with minimal side effects in healthy tissue. Recent studies have revealed that cancers with loss of function of *BRCA1* or *BRCA2* show exquisite sensitivity to Poly(ADP-ribose) polymerase (PARP)-inhibitors [17], [18]. Given that MRE11 is involved in DNA DSB repair through the MRN-complex, loss of function of this complex through inactivating mutations might lead to sensitivity to PARP-inhibitors [19]. PARP1, a DNA repair enzyme, has been implicated in the repair of DSBs. PARP inhibition leads to apoptosis or senescence in cells where DNA repair by homologous recombination (HR) is impaired (synthetic lethality). For this study we used a potent selective PARP1-inhibitor BMN673, that has shown very encouraging results in phase I/II trials [20].

Here we show that MRN is frequently lost in EC, which leads to increased PARP inhibitor sensitivity. This may be exploited for treatment of patients with EC harbouring loss of the MRN-complex. The goal of this study is to show the frequency of loss of MRE11 and MRN-complex in EC and whether this leads to increased sensitivity to PARP-inhibitors exploiting *MRE11* as a potential synthetic lethal gene.

## Materials and Methods

### Tissue Microarrays

Tissue microarrays (TMAs) with formalin-fixed and paraffin embedded endometrial carcinomas were constructed previously [21]. Two cohorts from the Institutes of Surgical Pathology, University Hospitals Basel and Zurich (Switzerland) containing 339 (Basel-TMA) and 182 (Zurich-TMA) cancer samples were included in this study. Clinical and pathological characteristics were taken from the clinical databases and pathology records. Routine hematoxylin and eosin sections were performed for histopathological evaluation. The stage of tumours was assessed according to the International Federation of Gynaecology and Obstetrics (FIGO) and TNM staging system. Histological subtype and tumour grade were defined according to the WHO classification 2003. Follow-up data are known from 480 patients. The median follow-up time was 31.5 months (range, 1–184) for the Basel cohort, and 45 months (range, 1–124) for the Zurich cohort. Patients with localized disease were treated by hysterectomy and bilateral salpingectomy (with or without pelvic and paraaortic lymphadenectomy). Vaginal radiation therapy was postoperatively administered when invasion of the myometrium or tumour grade 3 was evident. The study was approved for both cohorts by the local scientific ethics committee (KEK-ZH-NR: 2010-0358). Baseline characteristics of patients with EC are summarized in Table 1.

**Table 1:** Immunohistochemical expression of MRE11, RAD50, NBS1-p95, and mismatch repair proteins.

Immunohistochemical expression	n (%)
**MRE11 (n = 430)**	
negative	132 (30.7)
positive	298 (69.3)
**RAD50 (n = 440)**	
negative	107 (24.3)
positive	333 (75.7)
**NBS1-p95 (n = 433)**	
negative	127 (29.3)
positive	306 (58.7)
**MSH2/MSH6 (n = 487)**	
negative	112 (23.0)
positive	375 (77.0)
**MLH1/PMS2 (n = 497)**	
negative	139 (28.0)
positive	358 (72.0)

### Immunohistochemistry

After antigen retrieval, the slides were incubated with the following antibodies: MRE11 (clone 31H4, cell signalling, no. 4847, 1∶500), RAD50 (13B3/2C6, Abcam limited, no. ab89, 1∶500), NBS1 (cell signalling, no. 3002, 1∶50), MLH1 (G168-15, PharMingen, Becton Dickinson, 1∶100), MSH2 (25D12, Novocastra Lab. Ltd, 1∶100), PMS2 (A16-4, PharMingen, Becton Dickinson, 1∶300), and MSH6 (44, BD Biosciences, 1∶500). After incubation for 1 hour at room temperature, the staining of MRE11, RAD50, and NBS1 was further conducted with the Ventana Benchmark automated system (Ventana Medical Systems, USA) using Ventana reagents as well as UltraMap DAB detection kit. The antibodies against the mismatch repair proteins were incubated for 30 minutes and the staining procedure was carried out with the automated Leica BOND system using Bond Polymer Refine Detection Kit (Leica Biosystems). The expression analysis was performed by two pathologists (AN, KI). The nuclear immunoreactivity of MRE11, RAD50, and NBS1 was scored as: negative (0), weak (1), moderate (2) and strong (3). The protein expression of the mismatch repair genes was considered as positive when nuclear staining was evident. Stromal cells showing nuclear staining were used as a positive control.

### Cancer Cell Lines and Growth Conditions

The EEC cell lines Hec-108 [22] and Hec-116 [22] were grown in MEM, HEC 1A (ATCC) in McCoy’s, EFE-184 (DSMZ) in RPMI, AN3CA (ATCC) in DMEM, ARK-II [23] in DMEM, SNG-II [24] in DMEM, ECC-1 [25] in RPMI 1640, and were a gift from Uwe Schirmer, DKFZ. HEC6-ST3 [22], grown in MEM, and KLE (ATCC), grown in DMEM/F-12, were a gift from Jaqueline Galeas, UCSF. The media were supplemented with 10% (medium for ECC-1 with 5%) (v/v) fetal bovine serum and with either penicillin (100 U/ml)/streptomycin (60 mg/ml) or antibiotic-antimycotic, and maintained at 37°C in a humidified atmosphere at 5% CO2. All cell culture material was purchased from Gibco by LifeTechnologies.

### Immunoblotting

Whole-cell protein extracts were prepared from cells lysed with an SDS lysis buffer. Western blotting was performed with primary antibodies against MRE11 (D151, gift from Steve Jackson, Cambridge), RAD51 (rabbit polyclonal antibody, Santa Cruz Biotechnology), NBS1 (mouse monoclonal antibody, GeneTex), beta-tubulin (mouse monoclonal antibody, Sigma). Incubation with the primary antibody was followed by incubation with an HRP-conjugated secondary antibody (anti-mouse and anti-rabbit HRP from Sigma, anti-sheep HRP from SantaCruz) and chemiluminescent detection of proteins (Amersham).

### MRE11 Mutation Analysis

Thirteen MRE11 immunohistochemistry (IHC) positive and 13 IHC negative samples were chosen from the Zurich cohort for *MRE11* mutation analysis. Three tissue cylinders (diameter 0.6 mm) were punched from each paraffin block for DNA extraction. Genomic DNA was extracted using the DNeasy Blood & Tissue Kit (Qiagen, Hilden, Germany). The concentrations of the obtained nucleic acids were measured using the Nanodrop (Thermo Fisher Scientific, Waltham, MA, USA). PCR was performed as previously described (Grannini et al., 2002) with a slight modification. Only 50 ng of genomic DNA were PCR-amplified. DNA sequencing was carried out by using the BigDye Terminator v1.1 Cycle Sequencing Kit (Applied Biosystems). The obtained sequences were analyzed for mutations in the poly(T)11 repeat region within the human *MRE11* intron 4 (Table 2).

**Table 2:** MRE11 mutation analysis in endometrioid and serous endometrial carcinoma samples.

Sample No.	Histological subtype	p53 protein expression	MRE11 protein expression	MRE11 proteinintensity	MRE11 poly(T) alleles
en1	Endom	strong	positive	2	T_11_
en2	Serous		positive	3	T_11_
en3	Endom	neg	positive (low)	1	T_11_
en8	Endom	neg	negative	0	T_11_
en9	Endom	strong	positive	2	T_11_
en10	Serous		negative	0	T_11_
en11	Endom	low	negative	0	T_10–11_
en12	Endom	low	positive (low)	1	T_9–10_
en13	Endom	strong	positive	2	T_11_
en14	Endom	neg	negative	0	T_11_
en15	Endom	strong	positive	2	T_11_
en16	Endom	neg	negative	0	T_11_
en17	Endom	low	positive	2	T_11_
en18	Endom	neg	positive	3	T_11_
en19	Endom	low	positive	2	T_11_
en20	Endom	strong	positive (low)	1	T_11_
en21	Endom	low	negative	0	T_11_
en22	Endom	neg	positive (low)	1	T_11_
en23	Endom	neg	positive (low)	1	T_11_
en26	Endom	neg	negative	0	T_9_
en28	Endom	strong	negative	0	T_11_
en30	Endom	neg	negative	0	T_10–11_
en34	Endom	neg	negative	0	T_11_
en38	Endom	low	negative	0	T_10–11_
en40	Endom	neg	negative	0	T_11_
en62	Endom	strong	negative	0	T_11_

The immunohistochemistry status of p53 and MRE11 and the nuclear immunoreactivity of MRE11 are shown. Immunoreactivity of MRE11 was scored as: negative (0), weak (1), moderate (2), and strong (3).

### Colony Formation Assays

Long-term survival assays were performed as previously described [26]. In brief, cells were seeded in six-well plates in triplicates at a concentration of 300–2000 cells per well and treated with the inhibitor after 24 hours, being continuously exposed to the drug (10−11 to 10−6 M) dissolved in dimethyl sulfoxide (DMSO) (control: DMSO). Media and drug were replaced every 3 to 5 days. After 10 to 15 days, the cells were fixed with TCA 10% and stained with sulforhodamine B (SRB) (Sigma) and a colorimetric assay was performed as described previously [26]. The PARP inhibitor BMN673 was a gift from Biomarin Pharmaceuticals, USA. Mirin was purchased from Calbiochem.

### siRNA And Transfections

To knock down human *MRE11* we used three different siRNAs (Microsynth, Switzerland) using RNAimax (Gibco by LifeTechnologies) according to the manufacturer’s instructions. The target sequences (sense) were: GCUAAUGACUCUGAUGAUATT [27], GAGCAUAACUCCAUAAGUATT [28], and GAUGCCAUUGAGGAAUUAGTT [29]. As a control, cells were transfected with siRNA against luciferase (sense): CGUACGCGGAAUACUUCGATT. Cells were seeded 48 hours after transfection and treatment started 72 hours after transfection (PARP inhibitor, irradiation).

### Analysis of RAD51 Foci Formation

Nuclear RAD51 foci were visualized and quantified as previously described [26] and used as surrogate markers for induction of DNA DSBs and competent HR DNA repair, respectively. In brief, cells were grown on coverslips (12 mm, Thermo Scientific) and exposed to 10 Gy of IR. After 4–6 hours recovery, the cells were fixed, permeabilized, and immunostained with primary antibodies against RAD51 (polyclonal rabbit antibody, Santa Cruz) and detected with alexa flour 488 (Invitrogen). Nuclei were counterstained with 4′,6-diamidino-2- phenylindole (DAPI). The presence of RAD51 foci was evaluated in a minimum of 100 cells in three independent experiments with an automated inverted fluorescence microscope (LEICA–DMI6000 B).

### Statistical Analysis

The statistical evaluation was performed with the SPSS software Version 21.0 (SPSS Inc., Chicago, IL, USA). The scoring data of MRE11, RAD50, and NBS1 were dichotomized into “negative” (no expression) and “positive” (weak, moderate, or strong expression). The statistical significance of the association between these molecules, and the mismatch repair protein expression as well as clinicopathological parameters was assessed by Chi^2^ test for trends and Fisher’s exact test. In addition, Spearman’s correlation was used to evaluate an association between MRE11, RAD50, NBS1, and mismatch repair proteins. The probability of overall survival as a function of time was determined by the Kaplan-Meier method. Differences in the survival curves were compared by the log rank test. Sensitivity curves were calculated in GraphPad Prism Version 6 (GraphPad Software Inc., La Jolla, USA) and statistical differences between IC50 values were assessed using F-tests. Generally, p-values smaller 0.05 were considered as significant.

## Results

### MRN-complex Expression and the Association with Mismatch Repair Protein Status and Clinicopathological Factors

**Figure 1:**
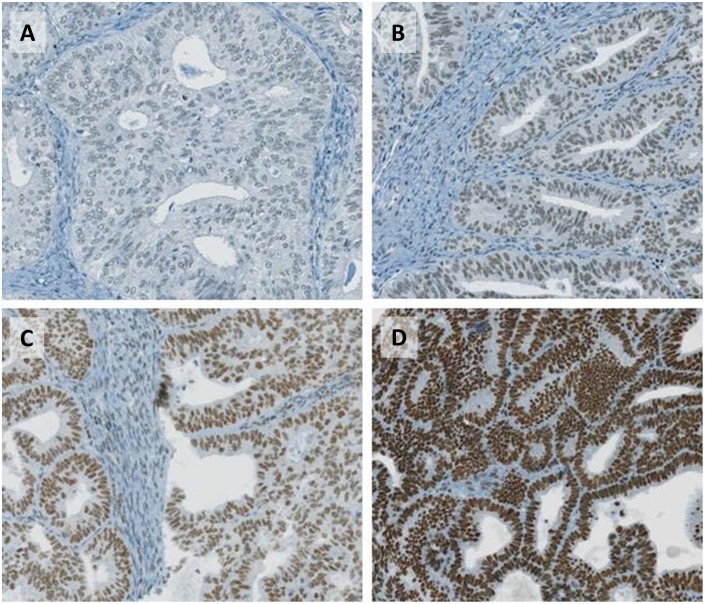
MRE11 protein expression in endometrial carcinomas as assessed by immunihistochemistry. A, negative (complete loss of MRE11 protein expression); B, + (weak nuclear expression); C, ++ (intermediate nuclear expression); D, +++ (strong nuclear expression).

Immunohistochemical analysis of MRE11, RAD50, and NBS1 was successful in 456, 466, and 458 endometrial carcinomas, respectively, due to the lack of tumour tissue in some TMA spots. All molecules showed a nuclear staining pattern. Among the 521 endometrial carcinomas patients 30.7% (132/430) showed a complete loss of MRE11 (Fig. 1A, and Table 1). Expression loss of MRE11 significantly correlated with protein loss of the other MRN-complex members RAD50 and NBS1. Loss of any MRN-complex protein was also significantly associated with mismatch repair protein expression as well as with FIGO stage and histologic grading (Table 3). In Kaplan-Meier analysis, FIGO stage, histological subtype, grading and patient age were prognostic factors (log rank, p-value<0.0001 for all parameters). However, protein expression of MRE11 (log rank, p = 0.867), Rad50 (log rank, p = 0.986) and NBS1 (log rank, p = 0.991) was not associated with overall survival. Moreover, sequencing of the poly(T)11 allele of *MRE11* in genomic DNA from EC tumours (n = 26; Table 2) showed that mutations were more frequent in tumours with loss of MRE11 than in those expressing MRE11, but the mutation status was not highly predictive for the protein status of MRE11.

**Table 3:** Association of MRE11 with RAD50, NBS1, mismatch repair protein status, and clinicopathological factors.

Variable	Mre11 negative	Mre11 positive	p-value
**RAD50**			**<0.0001** *
negative	87	18	
positive	45	280	
**NBS1**			**<0.0001** *
negative	102	25	
positive	29	266	
**MSH2/MSH6**			**<0.0001** *
negative	73	26	
positive	54	249	
**MLH1/PMS2**			**<0.0001** *
negative	54	66	
positive	76	215	
**FIGO stage**			**0.009** **
I	55	160	
II	17	40	
III	29	42	
IV	6	8	
**Histologic subtype**			**0.69** **
endometrioid	113	248	
serous	3	28	
clear cell	6	7	
undifferentiated	6	6	
carcinosarcoma	4	9	
**Grading**			**0.019** **
1	60	159	
2	33	74	
3	31	41	

*Fisher’s Exact test;

**chi-square for trends.

### Loss of MRE11 is Associated with High Sensitivity to PARP Inhibition

**Figure 2:**
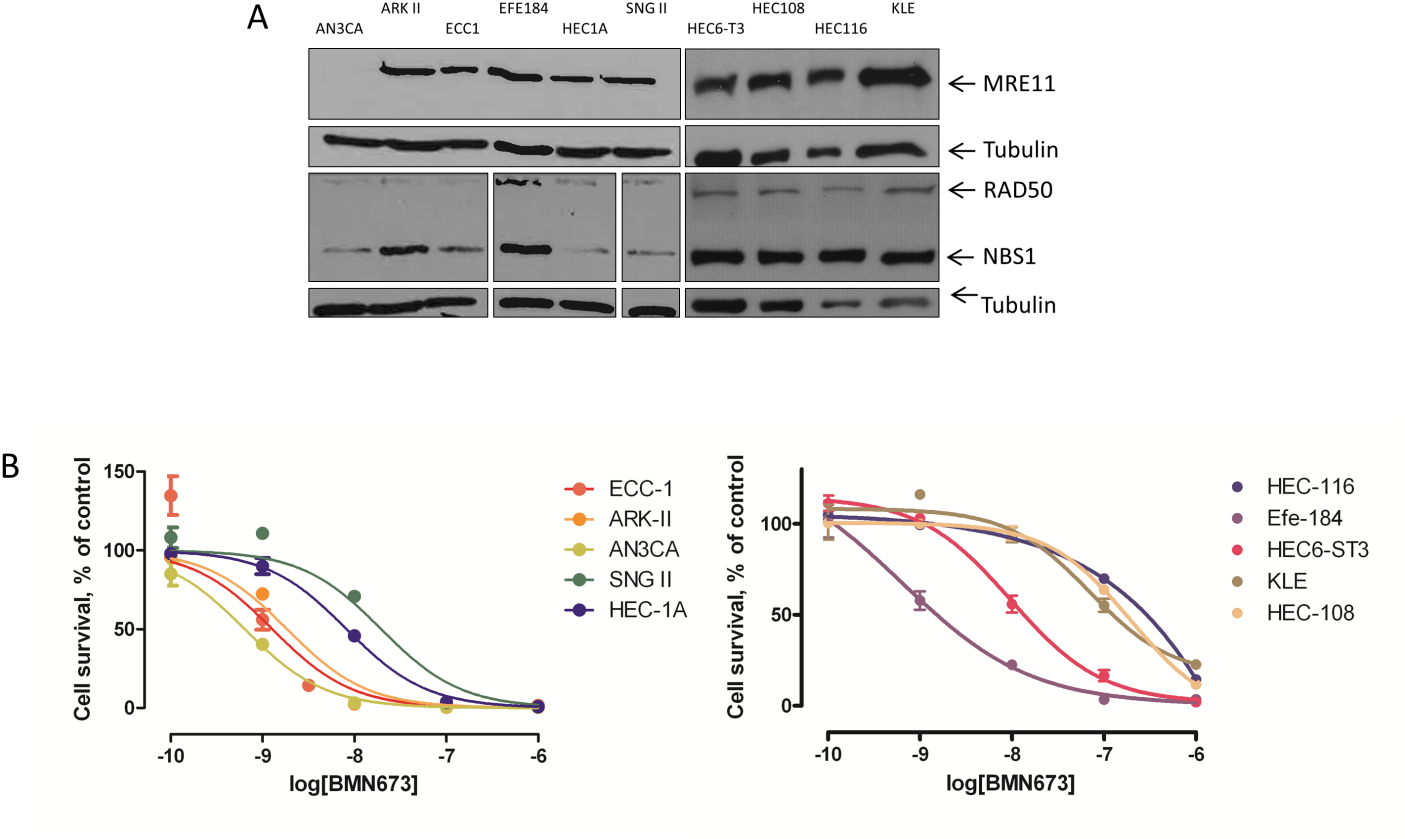
Endometrial carcinoma cell lines with loss of MRE11 show increased sensitivity to PARP inhibitors. A, Western blot demonstrating the expression status of MRE11, NBS1 and RAD50 in 10 endometrial carcinoma cell lines. Only the cell line AN3CA harbours a mutation of MRE11, which leads to a dramatically reduced expression of MRE11. Tubulin was used as loading control. B, Colony formation assays demonstrating the sensitivity to the PARP inhibitor (BMN673) in 10 endometrial carcinoma cell lines: all cell lines were treated in triplicates for 14 days. Survival is represented in percentage of the control (DMSO treated). The cell colonies were quantified using a colorimetric method (SRB).

**Figure 3:**
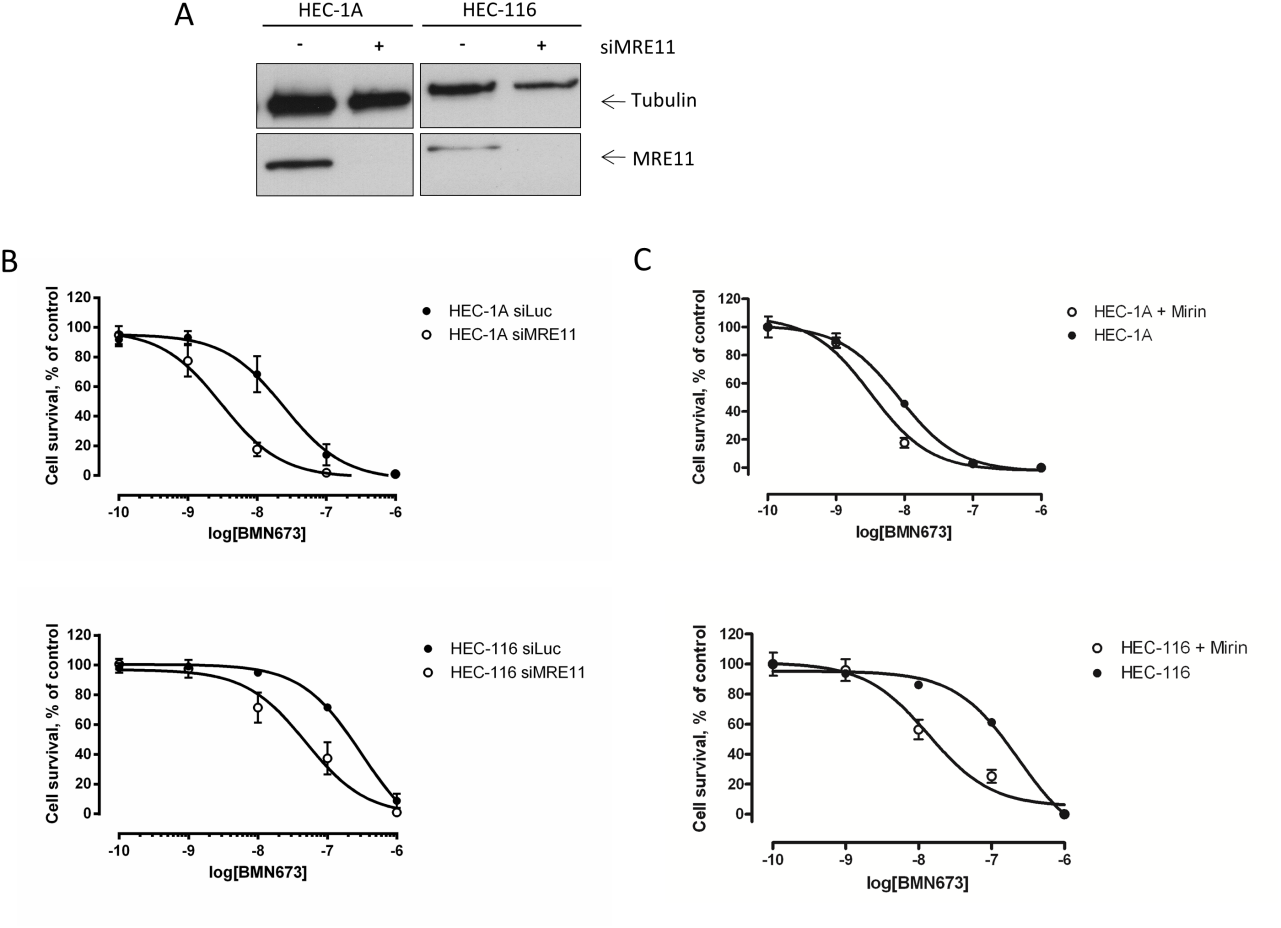
Decrease in MRE11 expression and function leads to increased sensitivity towards PARP inhibition in endometrial carcinoma cell lines. A, Efficiency of siRNAs at different time points after transfection shown by western blotting. B, Sensitivity assay (colorimetric with SRB): all cell lines were treated with PARP inhibitor (BMN673) in triplicates for 14 days. Survival is represented in percentage of the control (DMSO treated). Knock down of MRE11 with three different sets of siRNAs lead to an increased sensitivity towards PARP inhibition in the endometrial carcinoma cell lines HEC1A (8-fold increase, p<0.0001) and HEC-116 (6-fold increase, p = 0.0006). C, Cell lines were treated with the MRE11-inhibitor mirin at a final concentration of 30 µM in triplicates for 10 days. Survival was assessed by a colorimetric sensitivity assay (SRB) and is shown in percentage of the control (DMSO treated). Treatment with the MRE11-inhibitor mirin lead to increased sensitivity to PARP inhibition in both, HEC1A (2.6-fold increase, p = 0.001) and HEC-116 (17-fold increase, p<0.0001).

Among a panel of EC cell lines, AN3CA exhibited almost complete MRE11 protein loss as well as markedly reduced levels of RAD50 and NBS1 (Fig. 2A). Sequencing of the intronic region of MRE11 in this cell line confirmed the previously described homozygous poly(T)9 mutation in AN3CA (Fig. 2B) [11]. Upon PARP-inhibitor treatment with BMN673, AN3CA shows the highest sensitivity among the panel of endometrial cancer cell lines tested (Fig. 3C).

### Knock-down and Inhibition of MRE11 Sensitizes Cells to BMN673 due to Impaired HR

**Figure 4:**
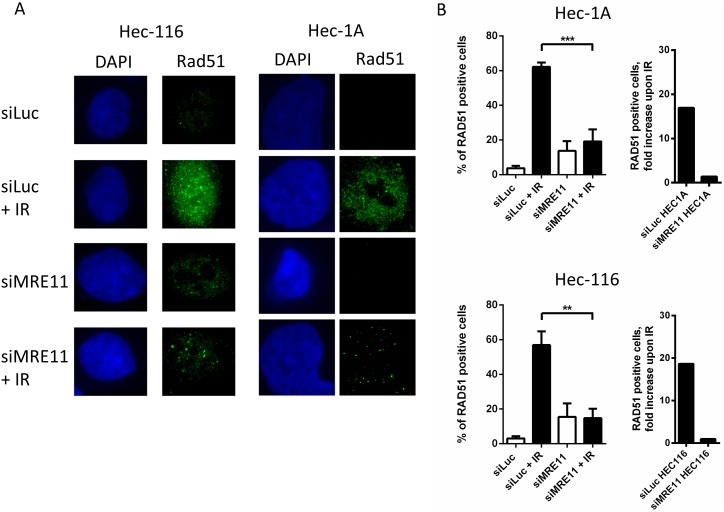
Impaired RAD51 foci formation upon knockdown of MRE11. A, Immunofluorescence images showing DAPI-stained nuclei (blue) and RAD51 foci (green) in representative fields of HEC-1A and HEC-116 endometrial carcinoma cells. Both wild-type MRE11 expressing cell lines were either treated with three different sets of siRNAs for MRE11 or with siRNA for luciferase (siLuc) as a control. The transfected cells were then exposed to 10 Gy of irradiation or not exposed, as indicated. B, RAD51 foci formation shown as a percentage of RAD51 foci positive cells (more than 5 foci per cell were assessed as positive) without treatment and 6 hours after 10 Gy of irradiation. A minimum of 100 cells was counted to determine RAD51 foci formation frequency in three independent experiments. **P≤0.01, ***P≤0.001.

Since loss of MRE11 expression in AN3CA was associated with significant sensitivity to PARP inhibition, we asked if depletion of MRE11 by siRNA would generally lead to an increased sensitivity to the PARP-inhibitor BMN673. To test this we used a panel of EC cell lines that expressed normal levels of MRE11 (Fig. 2A) and were also resistant to PARP inhibition (Fig. 2C). The efficiency of the siRNA treatment was evaluated by Western blotting. In both cell lines, transfection with MRE11 siRNA efficiently depleted endogenous MRE11 (Fig. 3A). We next assessed PARP inhibitor sensitivity in the MRE11-depleted cells by colony formation. Upon depletion of MRE11, we consistently observed a significant decrease in cell viability in the presence of BMN673 (Fig. 3B). In order to test if MRE11 nuclease activity is required for PARP inhibitor resistance, we cultured two cell lines in the presence of the MRE11 inhibitor mirin and treated them with BMN673 (Fig. 3C). In both cases mirin had a negative impact on the cell viability in response to PARP inhibition, strongly suggesting that MRE11 nuclease activity is required for PARP inhibitor resistance. To confirm that knock down of MRE11 leads to impaired HR, we determined the cells’ ability to form RAD51 foci in response to ionizing radiation (IR) (see Fig. 4A). Indeed, upon silencing of *MRE11,* fewer RAD51 foci per cell could be observed (Fig. 4B). The Capan-1 pancreatic cell line known to be HR-deficient due to a mutation of *BRCA2* served as a control (data not shown).

## Discussion

This is the first report to show that not only the protein expression of MRE11 but also the expression of the other members of the MRN-complex, RAD50 and NBS1, are lost in a substantial proportion of the ECs. Furthermore, we observed a significant association between protein loss of all MRN members as well as mismatch repair protein status in this large dataset. Protein expression of the MRN-complex has not yet been studied in EC tumours. In bladder cancer, MRE11 has been shown to exhibit predictive properties for radiotherapy treatment [30]. Furthermore, complete loss of MRN-complex in MSI positive colorectal cancers is a frequent event [31], whereas loss of MRE11 in breast tumours is found only in 9% of cases [32].

Although a clear correlation between mutational status and loss of protein expression could not be found in this study, it is remarkable that a high correlation of protein loss of all MRN members as well as MSI status was found in this large dataset. Confounding results on the mutational status of the MRE11 polyT(11) allele may be related to known difficulties in sequencing of the MRE11 polyT(11) allele due to mispairing of the polymerase enzyme [33].

A previous study revealed high frequency of alterations of DSB repair genes in MSI positive ECs, where MRE11 and RAD50 exhibited heterozygous and homozygous mutations in 51% and 17%, respectively, without examining the impact of the loss of the proteins [6]. Mutations of the MRE11 polyT(11) allele are predominantly heterozygous mutations and it has been suggested that only those with mutations of two and more nucleotides as well as homozygous mutations have a functional impact in terms of loss of function [11], [12], [34], [35]. In this report, we cannot confirm the high frequency of intronic mutations but provide evidence that MRE11 protein is lost in a substantial proportion of ECs. Recently, it has been shown that whole exon sequencing of MRE11 revealed mutations in 1.9% (2 of 107) of the EC tumours within the exons [36]. However, intronic mutations have not been assessed, explaining why the frequency of *MRE11* mutations is reported to be low not only in the study by Price et al. [36] but also in a recent one by The Cancer Genome Atlas Research Network [5].

PARP inhibitors have shown remarkable sensitivity in BRCA1/2-deficient tumour models *in vitro* as well as in clinical trials involving carriers of *BRCA1/2* germ line mutations. Further evolving evidence, however, suggests the potential for a broader scope for PARP inhibitor activity [16]. In fact, for EC we have previously proposed loss of PTEN expression as a potential biomarker for the treatment with PARP-inhibitors based on preclinical data [26] as well as on a clinical case report [37]. Other studies however, have been questioning the role of *PTEN* in HR, suggesting that this might be a cell line specific phenomenon [38], [39]. Nevertheless, the exact mechanism of the involvement of *PTEN* in HR DNA repair remains to be elucidated.

Loss of MRE11 expression has been suggested to sensitize colorectal [35], [38], [40], breast [41] and haematological [42] cancer cell lines to PARP-inhibitors due to impaired HR DNA repair. Our report suggests, for the first time, the potential use of PARP inhibitors in the treatment of endometrial cancer based on preclinical findings. There is increasing evidence that patients suffering from endometrial cancer and not expressing MRE11 could be treated with BMN673. This supports the use of MRE11 as a predictive biomarker for PARP treatment.

In conclusion, this study shows that i) complete loss of the MRN complex is a frequent event in EC, ii) loss of MRE11 expression as well as gene silencing and pharmacological inhibition of the nuclease activity leads to sensitivity to PARP-inhibition *in vitro*, and iii) loss of MRE11 is associated with deficient HR DNA repair demonstrated upon irradiation. Based on these findings, we propose that MRE11 expression may be used as a potential predictive biomarker for the effectiveness of PARP inhibitor treatment in endometrial cancers with MSI.
